# High-Fat Diet Delays Liver Fibrosis Recovery and Promotes Hepatocarcinogenesis in Rat Liver Cirrhosis Model

**DOI:** 10.3390/nu16152506

**Published:** 2024-08-01

**Authors:** Daisuke Taguchi, Yohei Shirakami, Hiroyasu Sakai, Toshihide Maeda, Takao Miwa, Masaya Kubota, Kenji Imai, Takashi Ibuka, Masahito Shimizu

**Affiliations:** Department of Gastroenterology, Gifu University Graduate School of Medicine, Gifu 501-1194, Japan

**Keywords:** high-fat diet, liver fibrosis, cirrhosis, hepatocarcinogenesis, carbon tetrachloride

## Abstract

More effective treatments for hepatitis viral infections have led to a reduction in the incidence of liver cirrhosis. A high-fat diet can lead to chronic hepatitis and liver fibrosis, but the effects of lipid intake on liver disease status, including hepatitis C virus and alcohol, after elimination of the cause are unclear. To investigate the effects, we used a rat cirrhosis model and a high-fat diet in this study. Male Wistar rats were administered carbon tetrachloride for 5 weeks. At 12 weeks of age, one group was sacrificed. The remaining rats were divided into four groups according to whether or not they were administered carbon tetrachloride for 5 weeks, and whether they were fed a high-fat diet or control diet. At 12 weeks of age, liver fibrosis became apparent and then improved in the groups where carbon tetrachloride was discontinued, while it worsened in the groups where carbon tetrachloride was continued. Liver fibrosis was notable in both the carbon tetrachloride discontinuation and continuation groups due to the administration of a high-fat diet. In addition, liver precancerous lesions were observed in all groups, and tumor size and multiplicity were higher in the high-fat diet-fed groups. The expression of genes related to inflammation and lipogenesis were upregulated in rats fed a high-fat diet compared to their controls. The results suggest that a high-fat diet worsens liver fibrosis and promotes liver carcinogenesis, presumably through enhanced inflammation and lipogenesis, even after eliminating the underlying cause of liver cirrhosis.

## 1. Introduction

Liver cirrhosis caused by viral infections is decreasing due to treatments such as suppression of hepatitis B virus (HBV) activity and elimination of hepatitis C virus (HCV) [1]. Non-viral cirrhosis caused by alcohol and metabolic dysfunction-associated steatohepatitis (MASH) has been found to be on the rise [2]; however, HBV and HCV remain the main causes of liver cirrhosis [2]. In recent years, HCV treatment with oral medication has become possible in both compensated and decompensated cirrhosis, and the condition of cirrhosis after viral elimination has received increased attention. Accordingly, the inhibitory effect of anti-HCV therapy on the development of liver cancer has also attracted attention.

Liver cancer ranks fourth for mortality, accounting for 8.3%, of all cancer deaths worldwide, and the number of patients with this disease is expected to increase [3,4]. Hepatocellular carcinoma (HCC), which accounts for the majority of liver cancers, is known to develop in livers with persistent inflammation, including chronic hepatitis and liver cirrhosis. In livers without advanced fibrosis, the elimination of HCV can reduce the development of HCC, whereas in advanced liver cirrhosis, the elimination of the HCV virus does not reduce the risk of HCC development [5].

Steatosis, also known as fatty liver, is the initial stage of liver disease with an abnormal accumulation of fat within hepatocytes. Steatohepatitis is a more advanced stage of liver disease characterized by both fat accumulation and inflammation in the liver. Fibrosis is the stage of liver disease with excessive accumulation of extracellular matrix due to chronic inflammation. Recently, the incidence of hepatic fibrosis was reported to be closely associated with obesity, which mainly involves MASH [6,7]. Hepatic fibrosis leads to liver cirrhosis and subsequently HCC, and approximately 33% of patients with MASH are considered to have liver fibrosis [8]; therefore, strategies for the amelioration of chronic liver damage and steatosis are important for reducing the risk for liver fibrosis and improving the prognosis of patients with chronic hepatic diseases.

Liver cirrhosis, as well as the related HCC, are not caused by a single factor alone, but are often influenced by a variety of factors, including diet. Nutritional status is considered to have an influence on hepatic disease, and nutraceuticals can improve the prognosis of patients with liver cirrhosis [9,10,11]. Therefore, nutritional therapy is important even when liver cirrhosis is not caused by diet or alcohol. Among the nutritional therapies for liver cirrhosis, the effectiveness of branched-chain amino acids and late-evening snacks has been mentioned in Japanese clinical practice guidelines [11,12,13,14]. In addition, to minimize nocturnal fasting time, an early breakfast and late-evening snack have been recommended by the American Association for the Study of Liver Diseases practical guidance [15].

Several studies have reported the influence of nutrients on the prevention and treatment of hepatic diseases, which include that a high-fat diet (HFD) induces hepatic fibrosis in a rodent model of liver cirrhosis; however, there are no reports examining the effects of excessive lipid intake on liver cirrhosis after eliminating the cause. The aim of this study was to investigate whether consuming a HFD affects hepatic fibrosis and cirrhosis as well as changing the hepatic disease condition after the underlying cause was removed. We used a rat cirrhosis model caused by carbon tetrachloride (CCl_4_). Since hepatic tumor development was observed at the end of the study, the effect of a HFD on liver carcinogenesis was also evaluated.

## 2. Materials and Methods

### 2.1. Animals, Diets, and Chemicals

Twenty-four 6-week-old male Wistar rats were obtained from Japan SLC, Inc. (Shizuoka, Japan) and kept in plastic cages in a specific pathogen-free animal facility under controlled conditions of light (12/12 h light/dark cycle), temperature (23 ± 2 °C), and humidity (50 ± 10%). All rats were given free access to drinking water and food. Control chow diet of CRF-1 and HFD with 62.2% of the calories derived from fat was purchased from Oriental Bio Service (Kyoto, Japan) and the ingredients of the diet are shown in Table 1. CCl_4_ was purchased from WAKO and mixed 1:1 with the solvent corn oil and administered by gavage.

### 2.2. Procedure for Animal Experiments

**Figure 1:**
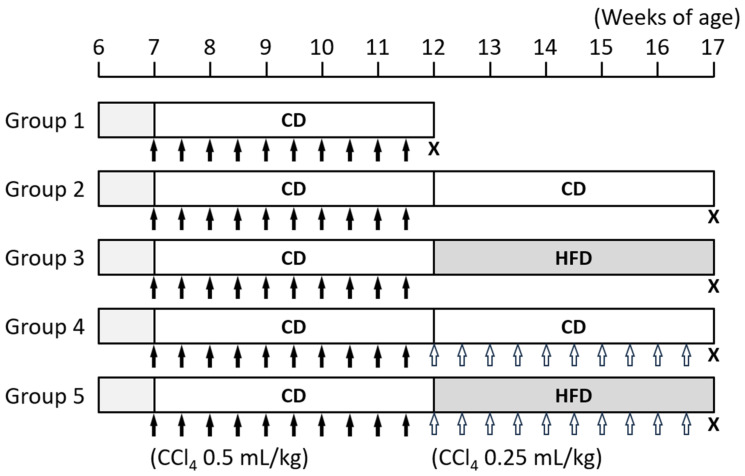
The protocol of this study. Male 6-week-old Wistar rats were randomly divided into five groups. In all groups, carbon tetrachloride (CCl_4_, 0.5 mL/kg, black arrow) was administered orally twice a week for 5 weeks to induce liver cirrhosis. The rats were then divided into the following groups: Group 1, the rats were sacrificed at 12 weeks of age; Group 2, the rats were fed CD without CCl_4_ for 5 weeks; Group 3, the rats were fed HFD without CCl_4_ for 5 weeks; Group 4, the rats were fed CD with oral administration of CCl_4_ (0.25 mL/kg, white arrow) twice a week for 5 weeks; Group 5, the rats were fed HFD with oral administration of CCl4 (0.25 mL/kg) twice a week for 5 weeks (*n* = 5). The rats in Groups 2 through 5 were sacrificed at 17 weeks of age. CCl_4_ was mixed 1:1 with corn oil and administered orally. CCl_4_, carbon tetrachloride; CD, control diet; HFD, high-fat diet; X, sacrifice.

The animal experiments were carried out with reference to a report by Iwasa et al. (Figure 1) [12]. After a week of acclimatization, the rats were randomly divided into five groups. A computer-generated randomization process was utilized to distribute the rats unbiasedly to the experimental groups. In all groups, CCl_4_ (0.5 mL/kg) was administered orally twice a week for 5 weeks to create a liver cirrhosis model. In Group 1, the rats were sacrificed at 12 weeks of age (*n* = 4). After the rats in Group 1 were sacrificed, the remaining rats were treated as follows. In Group 2, the rats received the control diet (CD) without CCl_4_ for 5 weeks (*n* = 5). In Group 3, the rats were fed HFD without CCl_4_ for 5 weeks (*n* = 5). In Group 4, the rats were fed CD with the oral administration of CCl_4_ (0.25 mL/kg) twice a week for 5 weeks (*n* = 5). In Group 5, the rats were fed HFD with the oral administration of CCl_4_ (0.25 mL/kg) twice a week for 5 weeks (*n* = 5). As shown in Figure 1, when comparing between Groups 2 and 3 as well as Groups 4 and 5, there was a clear distinction between the effects of the HFD in the presence or absence of CCl_4_. At 17 weeks of age, including 10 weeks of CCl_4_ administration, the rats in Groups 2–5 were killed after 12 h of fasting. To sacrifice the rats, CO_2_ inhalation was used. Approximately 2 mL of blood samples were collected from the inferior vena cava for chemical examinations, and organs and tissues were removed for histopathological examinations and gene expression analyses. The blood samples were kept at room temperature for 30 min and centrifuged at 1000× *g* for 20 min to obtain serum, which were then stored at −80 °C. We tried to minimize animal suffering in this study by providing an appropriate living environment for rats and establishing humane endpoints, which included the inability to eat or drink, weight loss (exceeding 20% compared to the baseline body weight or the control rats), persistent pain or distress, severe respiratory distress, and severe lethargy or immobility. The experimental protocol was approved by the Committee of Institutional Animal Experiments of Gifu University (approval code 2020-267).

### 2.3. Histology and Immunohistochemistry

The liver was fixed with 10% buffered formaldehyde and then embedded in paraffin. Azan staining and alpha-smooth muscle actin (αSMA) immunohistochemical staining were performed to assess hepatic fibrosis. To evaluate liver fibrosis, Azan-stained areas were imaged using NIH Image software version 1.62 (Bethesda, MD, USA) according to the tutorials and examples section on the software website (https://imagej.nih.gov/ij/docs/examples/stained-sections/index.html, accessed on 15 November 2023). Hematoxylin eosin (HE) staining and placental glutathione S-transferase (GST-P) immunohistochemical staining using primary antibodies against GST-P (MBL Co., Ltd., Tokyo, Japan) were performed to detect and evaluate hepatic precancerous lesions. The number of GST-P positive foci per rat was evaluated as multiplicity in each liver section using a light microscope. The size of each focus was measured and the maximum diameters of the lesions in each rat were compared.

### 2.4. Quantitative Real-Time Reverse Transcription-PCR

Total ribonucleic acid (RNA) was isolated from the liver of the experimental rats using the Pure Link RNA Mini Kit (Thermo Fisher Scientific, Waltham, MA, USA) with on-column DNase I-digestion. First-strand complementary deoxyribonucleic acid (cDNA) was synthesized from 2 µg of total RNA using a High-Capacity cDNA Reverse Transcription Kit (Thermo Fisher Scientific) and a T100 Thermal Cycler (Bio-Rad Laboratories, Hercules, CA, USA). Quantitative real-time reverse transcription-polymerase chain reaction (qRT-PCR) analysis was performed using specific primers targeting the genes for *Acta2*, *Ccl2*, *Fasn*, *Il1b*, *Il6*, *Serpin1*, *Srebf1*, *Tgfb1*, *Timp1*, and *Tnf*. Each sample was analyzed on a LightCycler 96 System (Roche Diagnostics, Basel, Switzerland) with FastStart Essential DNA Green Master (Roche Diagnostics, Basel, Switzerland) and the results were standardized by *Gapdh* amplified in parallel. Primer sequences used to amplify specific genes have been previously reported [16,17,18,19,20,21] and are shown in Table 2.

### 2.5. Measurement of Serum Parameters

Measurement of serum alanine aminotransferase (ALT), glucose, hyaluronic acid, and Type IV collagen concentrations were contracted to SRL, Inc. (Tokyo, Japan). Homeostasis model assessment insulin resistance (HOMA-R) was calculated from the measured insulin and blood glucose levels [22]. Diacron reactive oxygen metabolites (dROMs), indicating the degree of oxidation, and biological antioxidant potential (BAP), indicating antioxidant capacity, were measured using Free Carpe Diem (Diacron International srl., Grosseto, Italy). Relative oxidative stress was assessed using the ratio of dROMs to BAP.

### 2.6. Statistical Analysis

Fisher’s exact test was used to determine if there are nonrandom associations between two categorical variables in a contingency table and was utilized to compare the frequency of preneoplastic liver lesions. In other cases, to determine whether there are statistically significant differences between the means of four or five independent groups, after confirming normality with the Shapiro–Wilk test, comparisons were performed using a one-way analysis of variance. If the ANOVA showed significant differences, the Tukey–Kramer multiple comparison test was performed to compare the values between groups. A nonparametric statistical analysis was performed between groups using the Kruskal–Wallis test followed by the Steel–Dwass test. Data are presented as means ± standard deviation. A two-sided *p* value < 0.05 was considered statistically significant.

## 3. Results

### 3.1. General Observations

**Figure 2:**
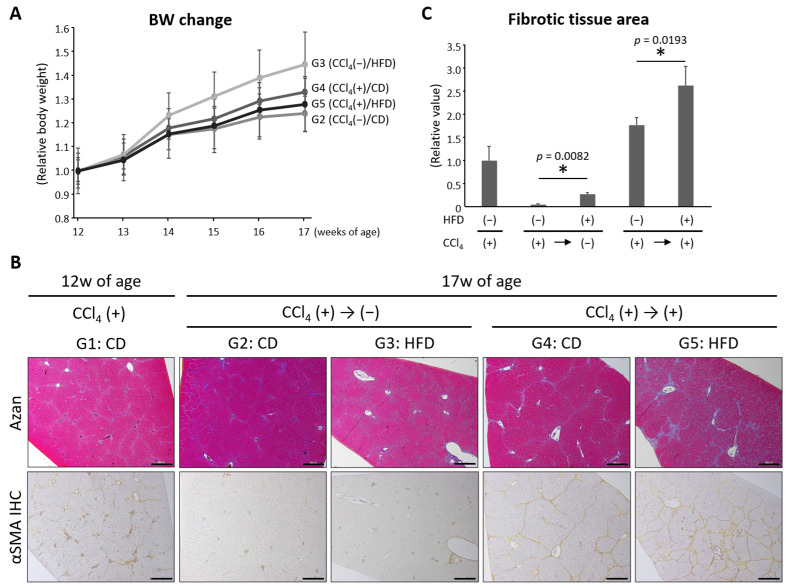
Body weight change and evaluation of hepatic fibrosis. (**A**) Body weight change of rats in Groups 2–5 (G2 to G5). (**B**) Representative pathological pictures of the liver. Azan staining (upper panels) and alpha-SMA immunohistochemical staining (lower panels) were performed for evaluating hepatic fibrosis. Rats in G1 were sacrificed at 12 weeks of age and the others at 17 weeks of age. Bars, 500 μm. (**C**) Quantitative analysis of hepatic fibrosis. Each column represents the mean ± SD. * *p* < 0.05. CCl_4_, carbon tetrachloride; CD, control diet; HFD, high-fat diet; IHC, immunohistochemical staining.

Regardless of the type of diet and CCl_4_ administration, rats in Groups 2–5 showed a trend toward increased body weight (Figure 2A). Rats in Group 3 that received HFD without continued CCl_4_, gained significantly more weight than those in Group 2 that received CD without CCl_4_ (Table 3). White adipose tissue was significantly heavier in rats in Group 3 than in Group 2.

### 3.2. HFD Exacerbated CCl_4_-Induced Liver Fibrosis and Delayed the Reversal of Fibrosis

**Figure 3:**
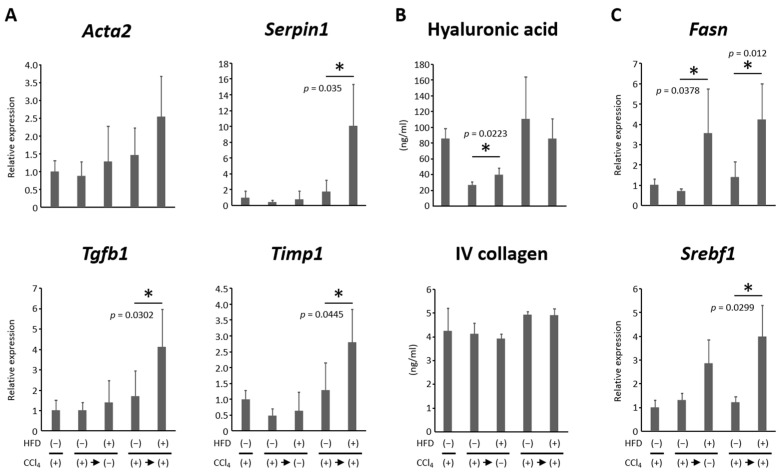
Effects of high-fat diet on hepatic fibrosis and steatosis in rat liver cirrhosis model. The expression levels of genes involved in (**A**) fibrosis, including *Acta2*, *Serpin1*, *Tgfb1*, and *Timp1*, and (**C**) lipogenesis, including *Fasn* and *Srebf1*, in the liver of experimental rats were examined by qRT-PCR using specific primers. The expression levels of each mRNA were normalized to the level of *Gapdh*. (**B**) Serum levels of hyaluronic acid and Type IV collagen were measured. (**C**) Each column represents the mean ± SD. * *p* < 0.05. *Acta*, actin alpha; CCl_4_, carbon tetrachloride; *Fasn*, fatty acid synthase; HFD, high-fat diet; *Serpin*, serine protease inhibitor; *Srebf*, sterol regulatory element binding transcription factor; *Tgf*, transforming growth factor; *Timp*, tissue inhibitors of metalloproteinase; qRT-PCR, quantitative real-time reverse transcription-polymerase chain reaction.

As previously reported [12], fibrosis formed in the liver after 5 weeks of treatment with CCl_4_ (Figure 2B). Liver fibrosis improved after the discontinuation of CCl_4_ in Groups 2 and 3, but fibrosis persisted to a greater extent in the HFD-fed rats than in the CD-fed rats (Figure 2B,C). Due to the additional 5 weeks of CCl_4_ administration, fibrosis and steatosis became more prominent in the liver of HFD-administered rats, and regenerative nodules surrounded by fibrous bands were evident in Group 5 (Figure 2B,C). The expression levels of genes involved in fibrosis in the liver as well as the serum levels of hyaluronic acid and Type IV collagen were also examined (Figure 3). *Serpin1*, *Tgfb1*, and *Timp1* levels significantly increased and *Acta2* levels tended to be up-regulated in the CC14-administered and HFD-fed groups compared to the CD-fed group (Figure 3A). Serum hyaluronic acid levels were higher in the CCl_4_-discontinued and HFD-fed rats than those in the CD-fed rats, while levels of Type IV collagen showed no significant difference between the CD- and HFD-fed groups (Figure 3B). These results suggest that HFD delayed the reversal of liver fibrosis in the CCl_4_-discontinued groups and exacerbated fibrosis in the CCl_4_-continued groups.

### 3.3. HFD Increased Lipogenesis and Induced Steatosis in the Liver

As expected, hepatic steatosis was induced by HFD administration in both the CCl_4_-discontinued and continued groups (Figure 2B). The expression levels of genes involved in lipogenesis in the liver were also examined. It was found that *Fasn* and *Srebp1c* were up-regulated in the HFD-fed groups compared to their control groups (Figure 3C).

### 3.4. HFD Promoted Hepatic Inflammation

**Figure 4:**
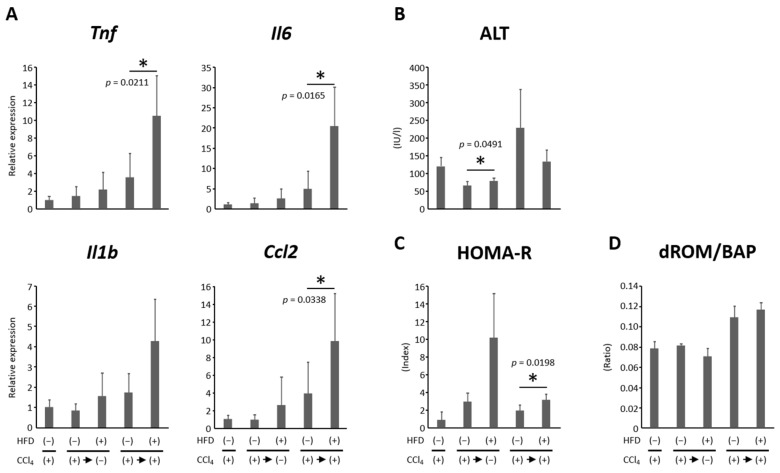
Effects of high-fat diet on inflammation in the liver, insulin resistance, and oxidative stress in rat liver cirrhosis model. (**A**) The gene expression levels of pro-inflammatory cytokines in the livers of experimental rats were examined by qRT-PCR using specific primers. The expression levels of each mRNA were normalized to the level of *Gapdh*. (**B**) Serum levels of alanine aminotransferase (ALT) were measured. (**C**) An indication of insulin resistance HOMA-R was assessed. (**D**) Oxidative stress was evaluated by the ratio of dROM to BAP in serum. Each column represents the mean ± SD. * *p* < 0.05. BAP, biological antioxidant potential; CCl_4_, carbon tetrachloride; *Ccl*, C-C motif chemokine ligand; dROM, diacron reactive oxygen metabolite; HFD, high-fat diet; HOMA-R, homeostasis model assessment insulin resistance; *Il*, interleukin; *Tnf*, tumor necrosis factor; qRT-PCR, quantitative real-time reverse transcription-polymerase chain reaction.

We also examined the gene expression of inflammatory cytokines in the liver. The levels of *Ccl2*, *Il6*, and *Tnfa* were significantly higher in the HFD-fed group than in the CD-fed group under CCl_4_ administration (Figure 4A). *Il1b* also tended to be higher in the HFD group although not significantly. Serum ALT levels were higher in the HFD-fed group than in the CD-fed group among the CCl_4_-discontinued groups, while the levels showed no significant difference among the CCl_4_-discontinued groups (Figure 4B). These results suggest that HFD administration exacerbated liver inflammation.

### 3.5. Effects of HFD on Insulin Resistance and Oxidative Stress

HOMA-R was assessed as an indication of insulin resistance and showed that it was higher in the HFD group both with and without continued CCl_4_ administration, and the difference was significant in CCl_4_-continued groups (Figure 4C). Although no clear difference was observed in the oxidative stress assessed by the ratio of dROMs to BAP, between the HFD and CD groups, the CCl_4_ continuation groups tended to have higher oxidative stress levels than those in the respective CCl_4_ discontinuation groups (Figure 4D).

### 3.6. HFD Promoted the Development of Hepatic Precancerous Lesions

**Figure 5:**
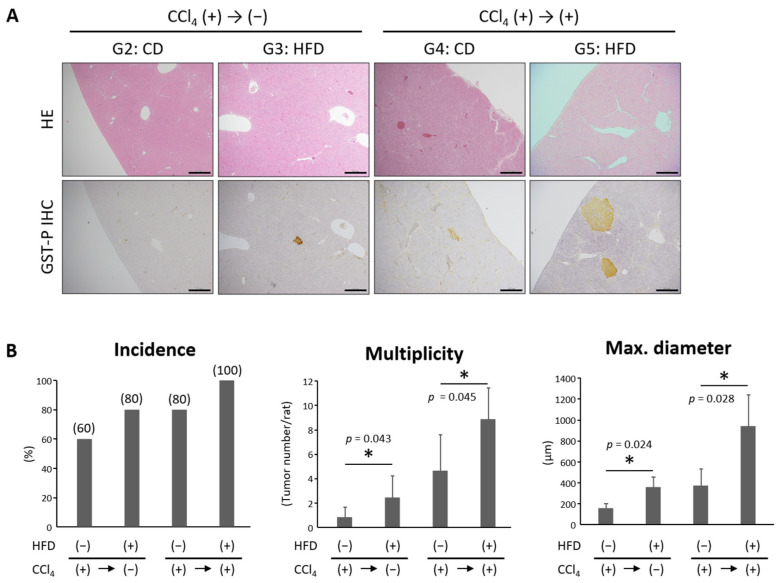
High-fat diet feeding promoted the development of hepatic precancerous lesions. (**A**) Representative pathological findings of HE staining and placental glutathione S-transferase (GST-P) immunohistochemical staining. Bars, 500 μm. (**B**) The incidence, multiplicity, and maximum diameter of hepatic tumors were assessed. Each column represents the mean ± SD. Significance is indicated as * *p* < 0.05. CCl_4_, carbon tetrachloride; HFD, high-fat diet.

Interestingly, precancerous lesions in the liver were observed in Groups 2–5 (Figure 5A). The incidence of the liver lesions tended to be higher in the HFD groups compared to the control CD groups, but the difference was not significant (Figure 5B). On the other hand, the variety and diameter of liver lesions in HFD groups were significantly higher.

## 4. Discussion

Liver cirrhosis caused by hepatitis viral infection has recently declined due to the development of treatments, including direct-acting antiviral drugs to eliminate HCV and nucleosides to suppress HBV activity. However, they remain a main cause of liver cirrhosis [1,2]. In addition, the development of liver cancer is also an important issue, even after the virus is eliminated or under control. Recent studies have shown that metabolic syndrome and obesity appear to be important risk factors for HCC [23]. Therefore, a nutritional approach is considered crucial for the control of chronic liver diseases and the prevention of liver cancer. In the present study, to investigate the effects of lipid intake on the control of liver cirrhosis, we administered HFD to a rat cirrhosis model. We found that HFD promoted CCl_4_-induced inflammation and fibrosis in the liver. We also confirmed that liver fibrosis improved after the removal of the fibrosis inducer CCl_4_, while HFD feeding delayed the reversal of fibrosis. In addition, precancerous lesions were observed in the liver, and HFD appeared to promote hepatocarcinogenesis independent of CCl_4_ continuity.

In the present study, liver fibrosis was observed after 5 weeks of CCl_4_ administration, and the fibrosis was then increased by a further 5 weeks of HFD feeding. Few reports have examined the effects of a HFD on CCl_4_-induced liver cirrhosis. HFDs have been used to create models of obesity and steatotic liver disease in rodents as well as models of MASH in combination with glucose and fructose or a low-choline and amino acid-defined diet [24,25]. A previous study showed that HFDs promoted hepatitis and led to obesity, with increases in body mass index of 1.5 times within a month, while only a trend toward liver fibrosis was observed after a month of HFD administration [26]. Other reports indicated that HFD feeding combined with a high cholesterol diet for 5 weeks was not sufficient time for liver fibrosis to occur [27], and that liver fibrosis occurred after 16 weeks when administering HFDs and one high-carbohydrate diet [24]. These reports suggest that inducing liver fibrosis with a HFD alone is difficult, at least for a relatively short period of time. In this study, the HFD promoted liver inflammation and insulin resistance, which may lead to an increased development of CCl_4_-induced fibrosis in the liver. This is consistent with the results of human studies where hepatic steatosis and insulin resistance contribute to fibrotic progression in chronic liver disease [28]. CCl_4_-induced liver fibrosis improves after discontinuation of its administration; however, rats given HFD showed a delayed reversal of liver fibrosis in this study. This could be due to the inflammation caused by steatohepatitis preventing liver fibrosis from improving.

As previously reported [29], this study also observed the development of CCl_4_-induced precancerous liver lesions promoted in the rats administered a HFD. The effects of HFDs on carcinogen-induced liver tumorigenesis in rats have been reported [30,31]; however, our study is believed to be the first report showing the effect of HFD on liver tumorigenesis after or during the administration of a carcinogenic agent. HFD feeding is known to induce inflammation and steatosis in the liver [24,32]. We found that serum transaminase as well as a hepatic expression of genes involved in inflammatory cytokines and lipogenesis increased in rats administered HFD. There is considerable evidence that steatotic liver disease increases chronic liver damage and leads to the development of liver cancer [33]. Furthermore, enhanced de novo lipogenesis is a common feature and an essential metabolic pathway in the pathogenesis of various cancers [34]. The expression of important enzymes for fatty acid synthesis is also reported to increase in several cancers, including liver cancer [35]. In the present study, HFD feeding up-regulated the gene expression levels of *Fasn* and *Srebp1c* in the liver, which may contribute to promoting the development of preneoplastic liver lesions. These results demonstrating the association between promoted liver tumor development and the increased expression of *Fasn* and *Srebp1c* are consistent with those of previous reports [36,37].

Although it remains unclear precisely how administering a HFD leads to hepatic fibrosis and HCC development, the accumulating evidence may indicate potential pathophysiological mechanisms, including insulin resistance, inflammatory cascades, and oxidative stress [38]. Insulin resistance is a major consequence of obesity as well as metabolic syndrome and causes fat accumulation and inflammation in the liver, which is considered MASH [39], and is known to worsen liver fibrosis and promote hepatocarcinogenesis [38]. Persistent hepatitis due to steatosis results in damage to hepatocytes, which leads to the activation of hepatic stellate cells and recruiting macrophages. Activation of hepatic stellate cells can promote the progression of MASH through the secretion of TNF-α, IL-6, and collagen, leading to inflammation and fibrosis in the liver [40]. In addition, insulin resistance also promotes the activity of insulin-like growth factor-1, an important factor for cell growth, proliferation, and survival. The alterations in insulin-like growth factor-1 and its receptor axis have been reported to contribute to the pathogenesis of liver cancer [41]. Moreover, insulin resistance increases circulating pro-inflammatory cytokines, such as TNF-α and IL-6, through exacerbated oxidative stress. The dysregulation of the above pro-inflammatory cytokines is considered to be related to steatohepatitis, liver fibrosis, and hepatic tumor formation [32]. In our study, the marker of insulin resistance was higher in the HFD-fed groups compared to their respective control groups, and differences were also noted in the degree of liver fibrosis and tumor development. These results are consistent with previous reports indicating the importance of insulin resistance for liver fibrosis and carcinogenesis. Oxidative stress in the liver increases with liver cirrhosis, and markers of oxidative stress in the blood are also increased in patients with chronic liver disease [42]. High-fat and high-cholesterol diets were also reported to worsen liver fibrosis in rodents through enhanced oxidative stress [27]. In addition, increased oxidative stress is associated with an increased risk of liver cancer [43]. However, in this study, we found no significant difference between the HFD- and CD-fed groups in terms of serum oxidative stress markers. The trend toward higher oxidative stress in the CCl_4_-continued groups compared to those in the CCl_4_-discontinued groups suggests that the enhancing effect of CCl_4_ on oxidative stress is stronger than that of HFD. DNA damage due to enhanced oxidative stress [44], an imbalance of adipocytokines from excess adipose tissues [45], an altered microbiome composition [46], and the regulation of autophagy [30,31] might also be caused by HFD feeding and contribute to promoted hepatic fibrosis and HCC development.

The HFD used in the present study was high-fat and low-carbohydrate and is referred to as the ketogenic diet. The ketogenic diet leads to the increased production of ketone bodies, which is an alternative energy source to glucose, and has been reported to possess the ability to treat cancer [47]. The beneficial effects of the ketogenic diet against cancer are explained by the fact that glucose is the essential energy substrate in cancer cell metabolism. While basic studies have provided several findings for the anti-cancer potential of the ketogenic diet, clinical trials for various malignancies have shown both promising and mixed results [47,48]. It was unclear whether the HFD used in our study had some effects on hepatic tumor development. Since the lesions developed in the liver were pre-cancerous, the HFD was thought to affect cancer initiation. It has been reported that the excessive accumulation of lipids promotes cancer initiation and the impact of lipid intake on carcinogenesis is associated with metabolic reprogramming and tumor microenvironment [49]. Further research is needed to investigate the effects of nutrient metabolism, including lipids, glucose, and ketone bodies, on hepatocarcinogenesis.

## 5. Conclusions

The present study indicates that the HFD exacerbated liver fibrosis and increased hepatic precancerous lesions even after eliminating the underlying cause of cirrhosis. The findings suggest that a fat-targeted diet and nutritional therapy may influence the pathogenesis of liver cirrhosis. Now, in clinical practice, the number of patients with chronic liver disease, whose underlying cause has been eliminated, is expected to increase [1]. In fact, it has been recognized that a proportion of patients are still at risk of developing HCC even after HCV elimination by direct-acting antiviral drugs; therefore, a reduction in the risk of HCC development and an examination of its molecular mechanisms, and the establishment of prediction and prevention methods are emerging as essential clinical issues. Investigations into pathways to recover from advanced liver fibrosis, which is considered to pose a high risk for liver carcinogenesis, are important as well. Since obesity and its associated metabolic disorders are serious health issues, which cause a variety of health-related complications, including cancer, weight management is needed. In addition, nutraceutical approaches, including the control of fat intake and taking antioxidant-rich foods, may be potential strategies for ameliorating chronic hepatic damage due to steatosis and for preventing liver cancer development and contribute to a better prognosis for patients with chronic liver disease even after the causative factors are removed.

## Figures and Tables

**Table 1:** Composition and calories of the experimental diets.

Ingredients (g/kg)	Control Chow	HFD
Casein	140	256
Corn starch	465.7	160
Sucrose	100	55
Dextrose	155	60
Cellulose	50	66.1
Soybean oil	40	20
Lard	0	330
Vitamin mixture	35	35
Mineral mixture	10	10
Calcium carbonate	0	1.8
L-cysteine	1.8	3.6
Choline bitartrate	2.5	2.5
Energy (kcal/g)	3.85	5.06

**Table 2:** Primer sequences.

Gene	Forward	Reverse
*Acta2*	CTGGAGAAGAGCTACGAACTGC	CTGATCCACATCTGCTGGAAGG
*Ccl2*	CGTGCTGTCTCAGCCAGAT	GGATCATCTTGCCAGTGAATG
*Fasn*	TCGTCTGCCTCCAGATCC	GGCAATTTCCCGGACATAC
*Gapdh*	TGGGAAGCTGGTCATCAAC	GCATCACCCCATTTGATGTT
*Il1b*	AGGCTTCCTTGTGCAAGTGT	TCCTGGGGAAGGCATTAGGA
*Il6*	CCCTTCAGGAACAGCTATGAA	ACAACATCAGTCCCAAGAAGG
*Serpin1*	CCCCGGCACTGGTAAATCTT	TCTGTCTATCTGCTGCCCCT
*Srebf1*	GGAGCCATGGATTGCACATT	GCTTCCAGAGAGGAGCCCAG
*Tgfb1*	CTTCAGCTCCACAGAGAAGAACTGC	CACGATCATGTTGGACAACTGCTCC
*Timp1*	ACAGCTTTCTGCAACTCGGA	AGTTTGCAAGGGATGGCTGA
*Tnf*	AGTTGGGGAGGGAGACCTT	CATCCACCCAAGGATGTTTAG

**Table 3:** General observation.

Group	Treatment	Number of Rats	Body Weight (g)	Relative Organ Weight (g/100 g Body Weight)	
				Liver	White Adipose Tissue
G2	CCl_4_ (−)/CD	5	367.2 ± 12.0 ^a^	2.8 ± 0.2	0.9 ± 0.3
G3	CCl_4_ (−)/HFD	5	438.4 ± 37.6 ^b^	2.8 ± 0.2	1.8 ± 0.4 ^b^
G4	CCl_4_ (+)/CD	5	372.2 ± 19.8	3.0 ± 1.5	0.8 ± 0.2
G5	CCl_4_ (+)/HFD	5	367.0 ± 34.4	3.0 ± 0.2	1.1 ± 0.3

^a^ Mean ± SD. ^b^ Significantly different from Group 2 (*p* < 0.05). CCl_4_, carbon tetrachloride; CD, control diet; HFD, high-fat diet.

## Data Availability

All data that supports the findings of this study is available within the article.
